# Cardiac effects of prolonged recombinant human interleukin-11 treatment in severe thrombocytopenia: a retrospective cohort study

**DOI:** 10.1186/s40360-026-01188-y

**Published:** 2026-07-20

**Authors:** Xian-fa Li, Yue Sun, Wen-rong Wang, Zi-yang Bao, Yong-zhong Zhong, Hong-yu Chen, Dong-rong Yu, Cai-feng Zhu, Jun Ni, Bin Zhu

**Affiliations:** 1 Department of Nephrology (Key Laboratory of Kidney Disease Prevention and Control Technology, Zhejiang Province), Hangzhou TCM Hospital Affiliated to Zhejiang Chinese Medical University, Tiyuchang Road 453, Hangzhou, Zhejiang Province 310007 China; 2 Department of Nephrology, Zhejiang Provincial People’s Hospital, Shangtang Road 158, Hangzhou, Zhejiang Province 310014 China

**Keywords:** Recombinant human interleukin-11, B-type natriuretic peptide, Arrhythmias, Thrombocytopenia

## Abstract

**Background:**

Recombinant human interleukin-11 (rhIL-11) is commonly used to treat thrombocytopenia induced by cancer chemotherapy. However, concerns about its potential cardiac side effects, especially with prolonged use, warrant further investigation.

**Aim:**

This study aimed to evaluate the cardiac effects of prolonged rhIL-11 use by comparing changes in cardiac biomarkers and arrhythmia rates between rhIL-11 and recombinant human thrombopoietin (rhTPO) in patients with severe thrombocytopenia.

**Method:**

We conducted a retrospective cohort study of adult patients treated with rhIL-11 or rhTPO for ≥ 5 days between 2015 and 2021. Data were collected at baseline, during treatment, and after discontinuation, focusing on BNP levels and arrhythmia occurrences. Statistical analyses were performed to compare the outcomes between the two groups.

**Results:**

Our study cohort comprised 23 patients treated with rhIL-11 and 7 patients receiving rhTPO. The rhIL-11 group showed a significant increase in serum BNP levels from a baseline median of 418 pg/mL (IQR: 193–1205) to 3084 pg/mL (IQR: 1446–5000) within one week (*P* < 0.01), while the rhTPO group had no significant changes (*P* = NS). BNP levels in the rhIL-11 group decreased to 384.0 pg/mL (IQR: 239.0–460.0) one week post-discontinuation (*P* < 0.05). Cardiac arrhythmias were documented in 34.8% (8/23) of rhIL-11-treated patients, demonstrating a clear treatment duration-dependent pattern: 83.3% (5/6) of patients receiving rhIL-11 for more than two weeks developed arrhythmias, with all 3 patients (100%) treated beyond three weeks experiencing cardiac complications, including two cases of frequent ventricular tachycardia. The rhTPO group showed no significant BNP changes or arrhythmic events. Kaplan-Meier survival analysis revealed a statistically significant divergence in arrhythmia risk profiles between the two treatment groups (*P* < 0.05). Multivariate COX regression analysis identified age as an independent risk factor for arrhythmia development (HR 1.071, 95% CI: 1.007–1.139; *p* = 0.029).

**Conclusions:**

Prolonged rhIL-11 treatment was associated with significant elevations in BNP levels and a higher incidence of arrhythmias, particularly with longer treatment durations. These findings highlight the need for close cardiac monitoring in patients receiving extended rhIL-11 therapy. Further large-scale, prospective studies are necessary to establish clear cardiac safety guidelines for rhIL-11 use.

**Supplementary Information:**

The online version contains supplementary material available at 10.1186/s40360-026-01188-y.

## Introduction

Severe thrombocytopenia is a critical hematologic condition that elevates bleeding risk, adversely affecting patient quality of life and placing a substantial burden on healthcare systems. Recombinant human interleukin-11 (rhIL-11) has been a therapeutic agent for this condition for decades, primarily due to its thrombopoietic activity. However, the biological understanding of interleukin-11 (IL-11) has undergone a paradigm shift. Initially characterized as a hematopoietic cytokine, IL-11 is now recognized as a pleiotropic factor with potent pro-fibrotic and pro-inflammatory properties across multiple organ systems, including the heart, kidney, and lung [1–4]. This duality is further complicated by the detailed description of IL-11’s pro-fibrotic mechanisms, which involve the activation of cardiac fibroblasts via ERK signaling and the induction of downstream microRNAs that serve as both mediators and potential circulating biomarkers of cardiac fibrosis [1, 5].

Clinically, rhIL-11 treatment is linked to cardiovascular adverse events such as fluid retention, atrial arrhythmias and transient ventricular dysfunction [6, 7]. Preclinical studies have uncovered the underlying molecular mechanisms: IL-11 directly binds cardiomyocyte IL-11 receptor α, triggers JAK/STAT3 stress cascades, disrupts intracellular calcium handling and causes acute left ventricular dysfunction [8]. Meanwhile, sustained IL-11 signaling promotes atrial fibrosis—the core pathological substrate of atrial fibrillation. Animal experiments further confirm that IL-11 blockade can reverse atrial fibrotic lesion [9, 10]. This fibrotic remodeling interacts with aging via age-dependent DNA methylation modification of IL-11 and its receptor, contributing to age-related cardiac injury [11].

Despite abundant mechanistic preclinical evidence, clinical data characterizing rhIL-11 cardiotoxicity, especially life-threatening ventricular arrhythmias observed in routine practice, remain insufficient. This study carries two urgent clinical rationales. First, rhIL-11 use is declining in many Western countries but remains a first-line supportive platelet-raising therapy in China. Given accumulating cardiac safety warnings, systematic re-evaluation of its long-term cardiac risk is urgently needed. Second, recent landmark studies defining IL-11 as a core pro-fibrotic mediator necessitate targeted clinical safety assessment for patients receiving prolonged rhIL-11 exposure.

To address this, we employed a retrospective cohort study design, leveraging a clinical database to evaluate the cardiac safety of long-term rhIL-11 use. The primary objective was to test the hypothesis that prolonged rhIL-11 administration leads to significant elevations in B-type natriuretic peptide (BNP), a marker of cardiac wall stress, and a higher incidence of arrhythmias. We further aimed to compare these outcomes with those in a control group receiving rhTPO, thereby isolating the effects specific to rhIL-11.

## Methods

### Patient selection

**Fig. 1:**
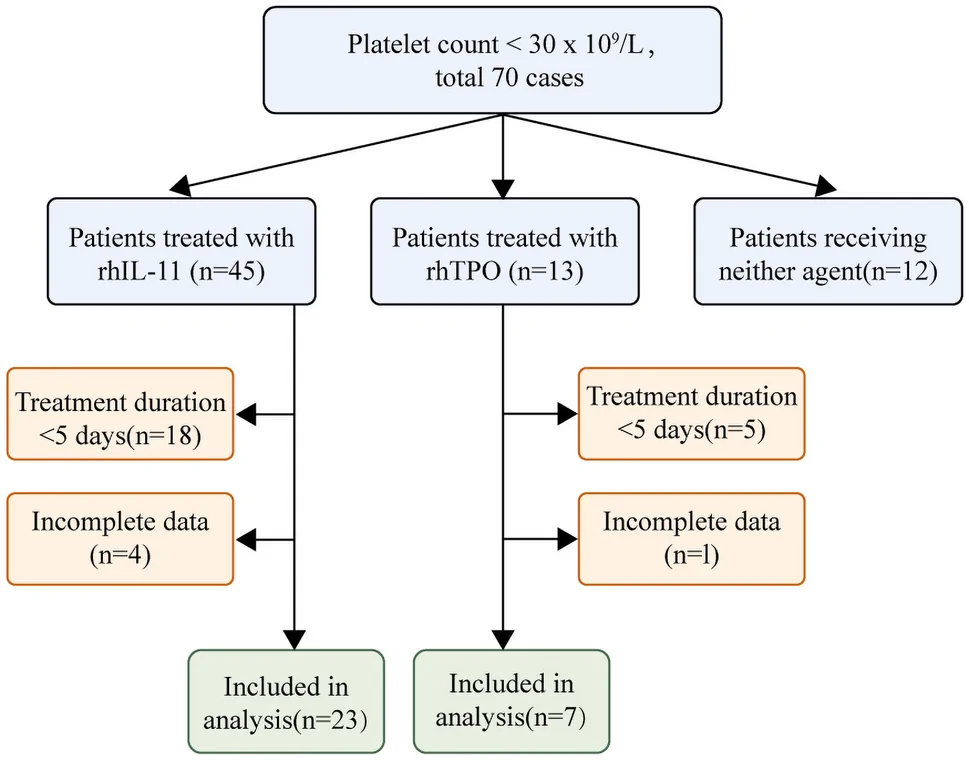
Patient screening flowchart

This retrospective single-center cohort study included adult patients with severe thrombocytopenia (platelet count ≤ 30 × 10⁹/L) treated with rhIL-11 or rhTPO for ≥ 5 consecutive days at the Department of Nephrology, Hangzhou TCM Hospital (July 2015–April 2021). Of 70 initial patients, 58 received either agent; after excluding those with treatment < 5 days (rhIL-11, *n* = 18; rhTPO, *n* = 5) or incomplete data (rhIL-11, *n* = 4; rhTPO, *n* = 1), 30 patients (23 rhIL-11, 7 rhTPO) were finally enrolled (Fig. 1). No patients were excluded on the basis of prior cardiac arrhythmias or other cardiac comorbidities. The study complied with the Declaration of Helsinki and was approved by the hospital’s Ethics Committee (No. 2022KY142, September 2022); informed consent was waived due to retrospective anonymized data, with all identifiers removed from the Hospital Information System(HIS).

Enrolled patients had thrombocytopenia from various etiologies (chemotherapy, aplastic anemia, idiopathic thrombocytopenia, etc.). rhIL-11 was given subcutaneously at 1.5 mg/day (25–50 µg/kg; lower dose used given lack of adjustment guidelines for renal impairment), and rhTPO at 15,000 U/day (~ 300 U/kg). Treatment was stopped upon reaching target platelet count (≥ 100 × 10⁹/L), hospital discharge (switching to oral agents), or adverse reactions (including cardiac events). No patient received concurrent chemotherapy during treatment. This study is reported in accordance with the Strengthening the Reporting of Observational Studies in Epidemiology (STROBE) guidelines for cohort studies (Supplementary File 1).

### Diagnosis and evaluations

Data collected included age, sex, treatment duration, BNP, electrolytes, cardiac enzymes, ECG, and echocardiography. BNP levels below 100 picograms per milliliter (pg/mL) were considered within the normal range, and a result of 5000 pg/mL was assigned to any tests exceeding the upper limit of 4989 pg/mL for statistical analysis. BNP was chosen over NT-proBNP because it is less affected by renal function—a relevant consideration given that renal insufficiency may elevate both markers, but NT-proBNP is primarily renally cleared and is more prone to accumulation in renal insufficiency. Therefore, BNP is a more suitable marker for assessing cardiovascular status in populations with renal impairment. Hypokalemia and hypocalcemia were defined as serum K⁺ <3.5 mmol/L and Ca²⁺ <2.02 mmol/L, respectively.

Given that patients with severe thrombocytopenia are often bedridden and susceptible to severe underlying conditions, there is a heightened risk of experiencing severe bleeding due to accidents. Consequently, certain factors such as lower extremity edema, body weight, and blood pressure were not accurately assessed during the study. However, it should be noted that none of the patients treated with rhIL-11 exhibited peripheral edema. As a result, the analysis included the factor of all-cause BNP variability. The primary endpoint event of this study was defined as the incidence of arrhythmia.

### Statistical analysis

GraphPad Prism 9.0 and SPSS 20.0 software (SPSS Inc, Chicago, Illinois) were utilized for statistical analysis. Data with a normal distribution were expressed as mean ± standard deviation, while data with an abnormal distribution were presented as median and interquartile range. The independent-samples T-test or paired-samples T-test was employed to assess statistical significance, unless the data exhibited an abnormal distribution. In such cases, either the Mann-Whitney U-test or the Wilcoxon signed-rank test was utilized. Kaplan-Meier survival analysis with a log-rank test was performed to compare survival rates. Cox proportional hazards regression models were employed for both univariate and multivariate analyses. The P value of less than 0.05 was considered statistically significant. For clarity and to avoid confusion regarding the direction of the inequality, all non-significant differences are denoted as “*P* = NS” throughout the manuscript. Due to the retrospective nature of this study, data for certain variables (including serial BNP measurements and echocardiographic parameters) were not routinely available for all patients at every predefined time point. Missing data were not imputed; all analyses used the available-case approach, with the number of available cases clearly indicated in tables and figure legends.

## Results

### Demographic characteristics

A total of 30 patients were enrolled in the study, including 23 patients in the rhIL-11 group (11 females and 12 males) and 7 patients in the rhTPO group (4 females and 3 males). The mean age was 59.2 ± 17.7 years in the rhIL-11 group and 56.1 ± 13.0 years in the rhTPO group, with no statistically significant difference between the two groups (*P* = NS; Table 1). The median treatment duration was 7.0 days (IQR: 5.0–17.0) in the rhIL-11 group and 8 days (IQR: 7–15) in the rhTPO group, showing no significant difference between the groups (*P* = NS; Table 1).

**Table 1:** Clinical characteristics of information in the rhIL-11 and rhTPO groups

Clinical characteristics	rhIL-11 group(*n* = 23)	rhTPO group(*n* = 7)	*p* Value
Sex (female/male)	11/12	4/3	1.00
Age (years, mean ± SD)	59.2 ± 17.7	56.1 ± 13.0	0.68
Usage time (days, median (interquartile range))	7(5,17)	8(7,15)	0.35
Number of new chest tightness (n (%))	5(21.7%)	0	
Time of onset of chest tightness (days, median (interquartile range))	8(5,17)	0	
Coma / dementia	5(21.7%)	0	
Number of arrhythmia cases (n (%))	8(38.1%)	0	
Time of onset of arrhythmia(days)	9.5 ± 6.3	0	
Number of electrolyte abnormalities (n (%))	7(30.4%)	3(42.9%)	0.66
Number of patients, whose usage time>14 days	6	2	1.00
Number of arrhythmia cases (n (%))	5(83.3%)	0	
CK at baseline (U/L, median (interquartile range))	43(23.3,74.0)	39.5(20.0,65.0)	0.74
CK at 1 week of medication (U/L, median (interquartile range))	42.0(22.0,110.0)	44.0(26.0,44.8)	0.761
* p1* Value	0.523	0.593	
CK-MB at baseline (U/L, median (interquartile range))	15.2(10.1,18.6)	15.8(11.9,20.1)	0.74
CK-MB at 1 week of medication (U/L, median (interquartile range))	14.6(8.1,17.9)	16.1(5.8,24.0)	0.94
* p2* Value	0.586	0.692	
LAD (mm)	34.8 ± 6.6	36.0 ± 3.5	0.69
LVEF (%)	64.4 ± 6.9	64.8 ± 5.8	0.90
Available Cases for BNP Analysis	10	4	
Sex (female/male)	6/4	2/2	1.00
Age (years, mean ± SD)	59.6 ± 19.0	54.5 ± 15.9	0.65

Notes: *p*1 is the comparison between the CK baseline and Week 1. *p*2 is the comparison between the CK-MB baseline and Week 1. LAD, left atrial diameter; LVEF, left ventricular ejection fraction

### Changes in clinical characteristics before and after rhIL-11 or rhTPO administration

During rhIL-11 treatment, 21.7% (5/23) of patients reported new-onset chest tightness by day 8 (IQR: 5–17) (Table 1). Notably, 5 patients in the rhIL-11 group had pre-existing conditions such as coma or dementia, which precluded their ability to report chest tightness. In the rhTPO group, 1 patient experienced chest tightness prior to treatment, accompanied by elevated BNP levels and hydrothorax, suggesting fluid overload. However, no new cases of chest tightness were observed in the rhTPO group during treatment. Additionally, no significant differences were observed in creatine kinase (CK) and its MB isoenzyme (CK-MB) levels between the two groups at baseline (*P* = NS; Table 1), after treatment (*P* = NS; Table 1), or in within-group comparisons before and after treatment (*P* = NS; Table 1). There were also no statistically significant differences in left atrial diameter (LAD) and left ventricular ejection fraction (LVEF) between the two groups (*P* = NS; Table 1).

### Changes in serum BNP levels before and after rhIL-11 or rhTPO administration

**Fig. 2:**
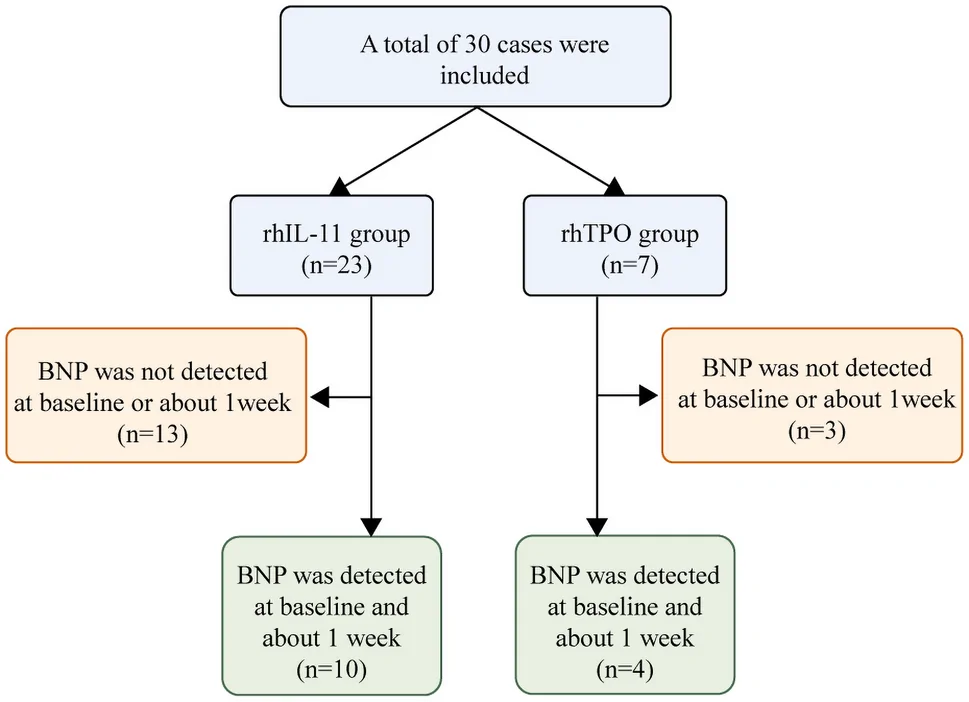
Flow chart of available cases of BNP affected by platelet-stimulating agents

**Fig. 3:**
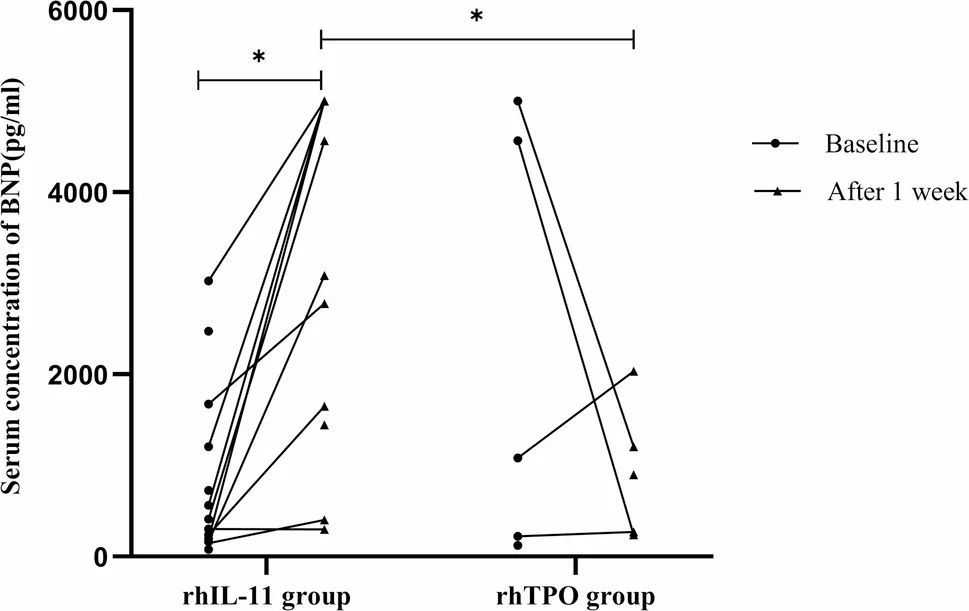
The variation of BNP after rhIL-11 or rhTPO administration. *P<0.01. Data are presented as median with interquartile range. Within-group comparisons before and after treatment were performed using the Wilcoxon signed-rank test. Between-group comparisons were performed using the Mann–Whitney U test. *P < 0.01

**Fig. 4:**
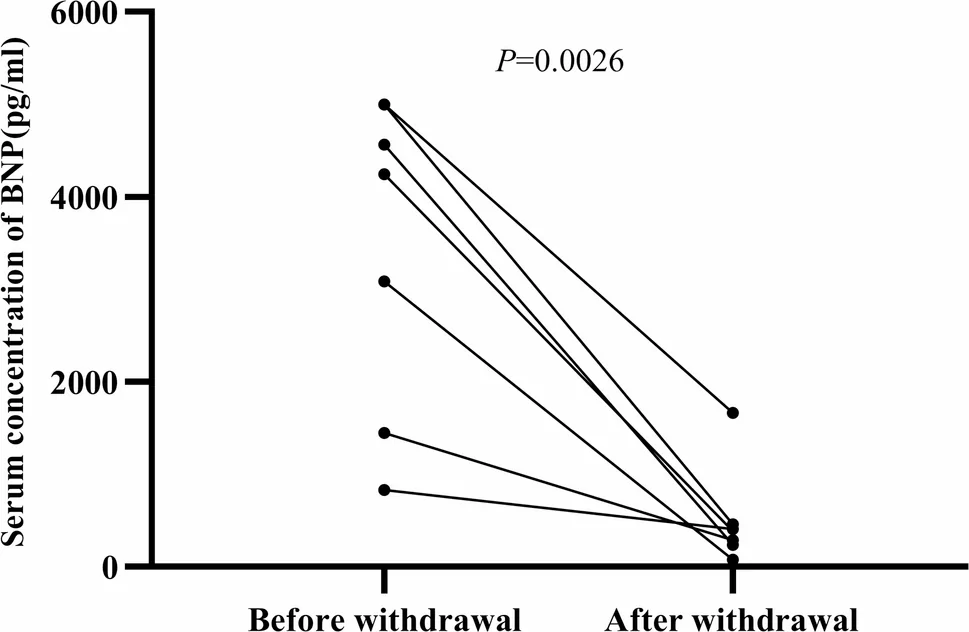
The variation of BNP before and after withdrawal of rhIL-11 (*P*<0.01). Data are presented as individual paired values with connecting lines. The comparison before and after withdrawal was performed using the Wilcoxon signed-rank test

Among the retrospectively enrolled patients, a total of 14 cases had BNP levels measured at baseline and approximately one week after treatment initiation (Fig. 2). Several other patients only had a single measurement. In the rhIL-11 group, the median serum BNP concentration was 418 pg/mL (IQR: 193–1205) at baseline. (Note: in the presence of renal dysfunction, BNP levels may be elevated due to reduced clearance; this should be taken into account when interpreting the values in this cohort.) During treatment, serum BNP levels significantly increased to a median of 3084 pg/mL (IQR: 1446–5000) within the first week (*P* < 0.01; Fig. 3). In contrast, the rhTPO group had a mean baseline BNP level of 1570.4 ± 2229.4 pg/mL, which decreased to 936.8 ± 858.4 pg/mL after approximately one week of treatment, with no significant difference between the two time points (*P* = NS; Fig. 3). While no difference in BNP levels was observed between the two groups at baseline, a significant difference emerged after one week of treatment, with the rhIL-11 group showing significantly higher BNP levels compared to the rhTPO group (*P* < 0.05; Fig. 3). Among the rhIL-11 group, BNP levels were also measured in seven patients before and after discontinuation of rhIL-11. The mean BNP level before discontinuation was 3452.9 ± 1718.0 pg/mL, which significantly decreased to a median of 384.0 pg/mL (IQR: 239.0–460.0) approximately one week after discontinuation (*P* < 0.05; Fig. 4).

## Effects of rhIL-11 or rhTPO on heart rhythm

Arrhythmias occurred in 8 out of 23 patients (34.8%) in the rhIL-11 group, with an average onset time of 9.5 ± 6.3 days after administration (Tables 1 and 2). In contrast, none of the 7 patients in the rhTPO group experienced any cardiac rhythm abnormalities. Among the 8 patients with arrhythmias in the rhIL-11 group, the observed rhythm disturbances included: 2 cases of sinus tachycardia (ST), 4 cases of atrial fibrillation (AF) or atrial flutter (AFL), 1 case of AF followed by frequent ventricular tachycardia (FVT), and 1 case of frequent premature ventricular contractions (FPVC) followed by FVT.

**Table 2:** Clinical features observed in 8 patients with arrhythmia in rhIL-11 group

Patients no.	Gender	Age (years)	Renal function status	Causes of Thrombocytopenia	Usage time (days)	BNP before medication (pg/mL)	BNP about 1 week of medication (pg/mL)	BNP about 1 week after withdrawal (pg/mL)	Electrolyte abnormalities	ECG at baseline	Time of onset of arrhythmia	Type of arrhythmia	Heart rhythm about 1 week after withdrawal
1	F	64	HD	SLE, APS	42	1674	2776	discharged	N	SR	AF on day 14, AFL on day 41	AF、AFL	NA
2	F	53	HD	Hypersplenism	37	725	NA	discharged	N	SR	AF on day 19,21, FVT on day 25	AF, FVT	NA
3	M	52	PD	Aplastic anemia	24	239	1650	411	N	SR	FPVC on day 13, FVT on day 24	FPVC, FVT	SR
4	F	53	HD	Hypersplenism	18	NA	NA	NA	hypokalemia	SR	14	ST	SR
5	F	83	HD	Drug-associated thrombocytopenia	17	3026	5000	384	N	SR	AF on day 5,9,14	AF	The AF did not recur. Amiodarone was switched to oral
6	F	85	Stage 5 CKD	Drug-associated thrombocytopenia	6	146	401	NA	hypocalcemia	SR	2	AF	SR
7	M	81	PD	Unknown causes	5	302.6	297	NA	hypokalemia	SR	3	ST	SR
8	F	56	Stage 5 CKD	Autoimmune thrombocytopenia	5	NA	1446	286	hypocalcemia	SR	6	TAFL	SR

Notes: N, normal; NA, not available; SR, sinus rhythm; AF, atrial fibrillation; AFL, atrial flutter; TAFL, transient atrial flutter; FVT, frequent ventricular tachycardia; FPVC, frequent premature ventricular contraction; ST, sinus tachycardia; ECG, electrocardiograph; HD, hemodialysis; PD, peritoneal dialysis; SLE, systemic lupus erythematosus; APS, antiphospholipid syndrome; CKD, chronic kidney diseases

In one patient (Case 2), AF occurred on day 19 and was successfully converted to normal sinus rhythm with medication. However, the patient experienced a recurrence of AF on day 21, followed by FVT on day 25, which ultimately progressed to ventricular fibrillation. In another patient (Case 3), FPVC developed on day 13, followed by FVT on day 24. The condition improved after treatment with amiodarone and multiple electrical cardioversions, with no recurrence observed one week after discontinuation of rhIL-11. Notably, electrolyte levels were within normal ranges during the arrhythmic episodes in both Case 2 and Case 3.

**Fig. 5:**
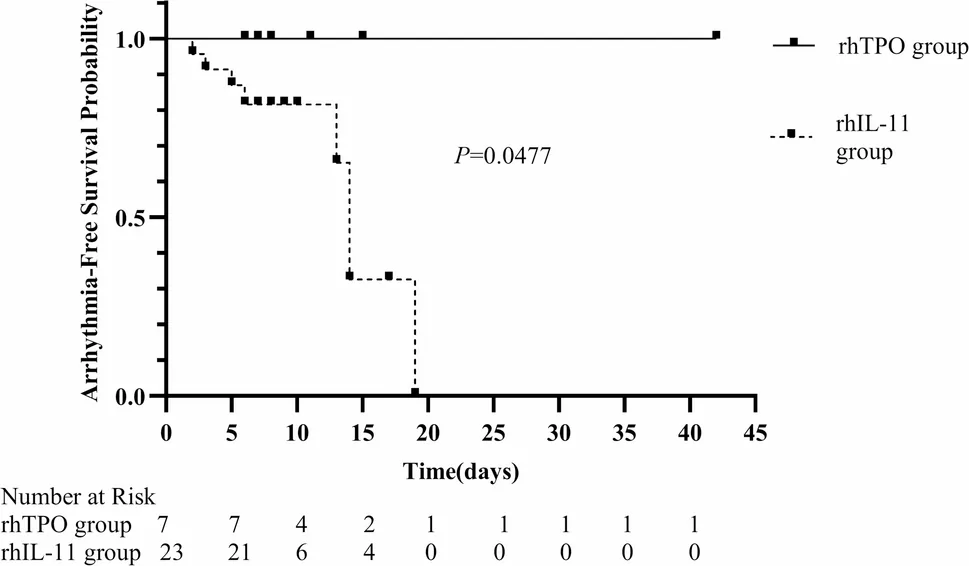
Kaplan-Meier survival analysis. The Kaplan-Meier curves depict the probability of remaining free from arrhythmia over the course of treatment. The dashed line represents patients in the rhIL-11 group (*n* = 23), and the solid line represents patients in the rhTPO group (*n* = 7). Tick marks on each curve indicate censored observations. The curves were compared using the log-rank (Mantel–Cox) test, which revealed a statistically significant difference in arrhythmia-free survival probability between the two treatment groups (χ² = 3.92, df = 1, *P* = 0.0477). The numbers at risk at each time point are displayed beneath the X-axis

Among the 8 patients with arrhythmias, 6 underwent heart rhythm monitoring one week after discontinuation of rhIL-11, and all returned to their baseline rhythm. In the rhIL-11 group, 6 patients received the drug for more than 14 days, and 5 of them developed arrhythmias. Among the 3 patients who received rhIL-11 for more than 21 days, all experienced arrhythmias, with 2 cases progressing to FVT. Electrolyte levels were measured during arrhythmic episodes in all 8 patients. Hypokalemia was observed in 2 patients with ST, and hypocalcemia was noted in 2 patients with AF or AFL. The remaining patients did not exhibit significant electrolyte abnormalities. A significant difference in arrhythmia-free survival probability was observed between the rhIL-11 and rhTPO groups (Log-rank test, Chi-square = 3.92, *df* = 1, *p* = 0.0477) (Fig. 5). In the univariate analysis, age demonstrated a borderline significant association with the outcome (*p* < 0.1), warranting its inclusion in the multivariate analysis. Furthermore, since the survival analysis revealed statistically significant differences between the rhIL-11 and rhTPO treatment groups, these variables were also incorporated into the multivariate Cox regression model. The multivariate analysis identified age as an independent risk factor for arrhythmia (HR 1.071; 95% CI 1.007–1.139; *p* = 0.029), as presented in Table 3. However, it should be noted that no endpoint events were observed in the rhTPO group, which consequently reduced the statistical power of the Cox model. This limitation resulted in wider confidence intervals for the hazard ratios and less precise estimates of the treatment effects.

**Table 3:** Univariate and multivariate analysis of independent prognosis factors for the primary endpoint event

Characteristics	Total(*N*)	HR (95% CI) Univariate analysis	*P* value	HR (95% CI) Multivariate analysis	*P* value
Sex	30				
Female	15				
Male	15	0.469 (0.090–2.445)	0.369		
Age (year)	30	1.056 (0.997–1.119)	0.062	1.071 (1.007–1.139)	0.029
Electrolyte abnormalities	30				
No	19				
Yes	11	2.078 (0.457–9.454)	0.344		
Group	30				
RhIL-11	23				
RhTPO	7	0.000 (0.000 - Inf)	0.999	0.000 (0.000 - Inf)	0.999*
LAD (mm)	25	1.043 (0.896–1.213)	0.589		
LVEF (%)	25	0.920 (0.788–1.075)	0.294		

Notes: *No patients in the rhTPO group reached the primary endpoint event, which reduced the statistical power of the COX model and resulted in wider confidence intervals for the hazard ratio, leading to less precise estimates. Given that the survival analysis demonstrated a statistically significant difference between the rhIL-11 and rhTPO groups, we included this comparison in the multivariate COX regression analysis. The primary endpoint event was defined as the occurrence of arrhythmia. LAD, left atrial diameter; LVEF, left ventricular ejection fraction

## Discussion

This study provides novel clinical insights into the acute and subacute cardiac effects of rhIL-11, challenging the perception that its cardiovascular side effects are sporadic or non-specific. Our findings demonstrate a robust association between prolonged rhIL-11 therapy and a dramatic surge in BNP levels coupled with a high incidence of arrhythmias. These observations are mechanistically grounded in an expanding body of preclinical evidence [1, 5, 8, 9].

The pronounced BNP elevation observed in our cohort, which peaked within the first week of treatment and returned to baseline shortly after drug discontinuation, is indicative of a direct and largely reversible cardiomyocyte stress response. This interpretation is consistent with the findings of Liu et al. [12], who documented a mild BNP increase (approximately 200 pg/mL) following rhIL-11 administration. The markedly higher BNP surge in our study—from a median of 418 pg/mL (IQR: 193–1205) to 3084 pg/mL (IQR: 1446–5000)—likely reflects the compounding effect of impaired renal clearance, a characteristic of our cohort predominantly composed of patients with chronic renal failure or those undergoing dialysis [13, 14]. Recent mechanistic studies have elucidated the cellular basis for this phenomenon, showing that IL-11 directly activates IL-11RA/JAK/STAT3 signaling in cardiomyocytes, thereby upregulating stress markers such as Nppb (the gene encoding BNP) and precipitating acute left ventricular dysfunction [8]. This direct cardiotoxic effect, converging with reduced BNP clearance in CKD, creates a scenario for profound BNP accumulation. The identification of BNP as a dynamic and responsive biomarker of rhIL-11-induced cardiac stress offers clinicians a valuable tool for therapeutic monitoring.

Our study further elucidates the time-dependent nature of cardiac arrhythmias, documenting an onset at 9.5 ± 6.3 days, aligning with prior reports [6]. A crucial finding is the dramatic escalation of arrhythmic risk with prolonged therapy. While the overall incidence was 34.8% (8/23), this rate rose to 83.3% (5/6) in patients treated for over two weeks, and reached 100% (3/3) in those receiving rhIL-11 for more than three weeks. The two latter cases, manifesting as ventricular tachycardia on treatment days 24 and 25, constitute a particularly alarming observation. Whereas atrial arrhythmias are a recognized complication of rhIL-11 therapy [12, 15], the occurrence of ventricular tachycardia represents a severe manifestation that has not been well characterized in prior clinical reports.

The mechanisms underlying these arrhythmias are likely multifactorial and time-dependent. In our study, patients who developed ventricular arrhythmias had normal serum electrolyte levels and myocardial enzymes, ruling out electrolyte disturbances and acute myocardial infarction as primary causes.

For atrial arrhythmias, the pathogenesis involves both acute and chronic components. Earlier electrophysiological studies excluded direct modulation of human atrial myocyte ion channels by rhIL-11 [16]. Instead, a two-hit model is supported: (1) an acute, indirect pathway wherein rhIL-11-induced sodium retention and volume expansion cause atrial stretch and refractory period shortening, facilitating re-entry [17]; and (2) a chronic, structural pathway driven by IL-11’s potent pro-fibrotic activity, as established in cardiac fibrosis models [1, 5, 9, 18–22].

However, arrhythmias in our cohort occurred after relatively short treatment durations (several days) and resolved rapidly upon drug discontinuation, arguing against fibrosis as the sole explanation, given that structural remodeling typically requires longer to develop and is not promptly reversible. More recent evidence reveals a direct cardiotoxic effect of IL-11 on cardiomyocytes via the IL-11RA/JAK/STAT3 pathway, leading to Nppb upregulation, disrupted calcium handling, and activation of pro-inflammatory transcriptional programs [8]. This direct myocardial toxicity—particularly calcium homeostasis perturbation—may independently create a pro-arrhythmic substrate and explain the worsening of arrhythmias with prolonged therapy.

Additionally, the predominance of ventricular arrhythmias in our cohort may partly reflect the high prevalence of underlying renal impairment. Chronic kidney disease is itself associated with latent ventricular remodeling, subclinical interstitial fibrosis, and altered ion channel/connexin expression, all of which create a vulnerable substrate for re-entry or triggered activity. When the direct cardiotoxic effects of rhIL-11 are superimposed on this pre-existing substrate, ventricular arrhythmias may be unmasked preferentially, even without electrolyte or enzymatic abnormalities.

Given the potentially life-threatening nature of ventricular tachycardia, these findings warrant heightened clinical vigilance and suggest the need for more intensive cardiac monitoring, particularly in patients receiving extended courses of rhIL-11 therapy beyond the two-week treatment period. In these patients, arrhythmias initially improved with pharmacological intervention during continued rhIL-11 administration but demonstrated a tendency for recurrence and progressive worsening over time. Among the observed cases, normal heart rhythm was restored following discontinuation of rhIL-11 treatment. Notably, all three patients with treatment durations exceeding three weeks maintained normal electrolyte profiles at the time of their arrhythmic events, indicating that the observed rhythm abnormalities were not driven by electrolyte disturbances. Furthermore, serial measurements of cardiac enzymes, including CK and CK-MB, revealed no significant fluctuations or pathological elevations throughout the treatment course, indicating the absence of acute myocardial injury in these patients. The Kaplan-Meier survival analysis demonstrated a statistically significant difference in arrhythmia risk between the rhIL-11 and rhTPO groups (log-rank *P* < 0.05). However, the absence of endpoint events in the rhTPO group limited the ability to accurately estimate hazard ratios between the two treatment groups. This study identified age as an independent risk factor for arrhythmia (HR = 1.071, 95% CI: 1.007–1.139; *P* = 0.029). Although the effect size is modest, it reached statistical significance. Of note, recent epigenetic research has revealed that DNA methylation of the IL-11 and its receptor genes exhibits a non-linear, inverted U-shaped change with age, peaking in middle-aged individuals (40–59 years), suggesting that the IL-11 pathway may be relatively suppressed during this period [11]. The mean age of our study population was 53.9 years, which falls exactly within this peak range, potentially explaining why the age-related risk increase was only modest rather than larger. Furthermore, animal model studies have confirmed that inhibiting IL-11 signaling significantly extends healthspan and mitigates age-related metabolic and muscle functional decline [23], indicating that IL-11-driven inflammation and fibrosis are core drivers of age-related pathology. Therefore, we hypothesise that age may promote low-grade myocardial inflammation and fibrosis—and consequently increase the long-term risk of arrhythmia—by influencing the epigenetic regulation and expression of inflammatory factors such as IL-11. Due to the relatively small sample size, the clinical predictive value of this HR estimate should be interpreted with caution. Nonetheless, when integrated with recent basic science findings, our results provide preliminary human evidence supporting further exploration of anti-inflammatory or anti-fibrotic strategies (e.g., targeting IL-11) for the primary prevention of arrhythmia in older adults. These findings carry immediate and practical implications for patient management. We advocate for careful cardiac monitoring for all patients receiving prolonged rhIL-11 therapy, with heightened vigilance for elderly patients and when treatment extends beyond two weeks. We recommend a comprehensive monitoring strategy that includes performing an electrocardiogram, measuring BNP levels, and monitoring serum electrolytes every two days, together with daily assessment for symptoms such as chest tightness or any cardiac discomfort. If any abnormalities are detected, we recommend immediately discontinuing rhIL‑11, switching to alternative platelet‑raising regimens (e.g., drug withdrawal, oral leukogen, rhTPO, or platelet transfusion), and consulting a cardiologist promptly.

Our study serves as a crucial clinical correlate to the extensive preclinical literature. While genetic and pharmacological inhibition of IL-11 has been shown to reverse organ fibrosis and extend healthspan in animal models [2, 3, 23], our data demonstrate that activating the same pathway with recombinant IL-11 is unequivocally harmful to the human heart. The reversible nature of these effects post-cessation mirrors preclinical observations where halting IL-11 expression resolves fibrosis and dysfunction [24, 25]. It is, however, important to acknowledge the broader biological context of IL-11, which has essential physiological roles in fertility and bone development [3] and is a critical mediator of epimorphic regeneration in species such as zebrafish [26, 27]. This highlights a fundamental biological dichotomy: a transient, localized pulse of IL-11 signaling is an integral component of tissue repair, whereas sustained, systemic, or supraphysiological activation—as is the case with therapeutic rhIL-11—is maladaptive and drives pathology. Our study captures this latter scenario, underscoring the dangers of chronic, systemic IL-11 pathway activation.

Despite these important findings, our study has several limitations that warrant consideration. The retrospective design, small sample size, and single-center nature are inherent weaknesses that limit the generalizability of our findings and their statistical power. The most significant analytical limitation arises from the comparison with the rhTPO group: the sample size was extremely small (*n* = 7) and, critically, no endpoint events (arrhythmias) occurred. This zero-event rate severely destabilized the multivariate Cox model, rendering the hazard ratio estimates for the treatment group comparison unreliable and leading to infinite confidence intervals, as shown in Table 3. Therefore, while the descriptive comparison is informative, the primary statistical conclusions should be drawn from the within-rhIL-11 group analyses (e.g., the impact of treatment duration and age), not the cross-group comparison. Overall, these factors limit generalizability, positioning the study as an exploratory analysis. Future, adequately powered prospective studies with balanced groups and predefined monitoring protocols are essential to validate and quantify this safety signal.

## Conclusions

Prolonged rhIL-11 treatment is associated with significant, reversible elevations in BNP and a substantial, duration-dependent risk of arrhythmias, including ventricular tachycardia. These findings provide clinical evidence of a direct cardiotoxicity that complements mechanistic discoveries from preclinical models. Our data strongly advocate for mandatory and intensive cardiac monitoring in any patient receiving rhIL-11 therapy, especially beyond two weeks. In light of these safety concerns and the availability of alternative agents, the risk-benefit profile of extended rhIL-11 use must be carefully re-evaluated. Large-scale, prospective clinical trials are urgently needed to establish definitive cardiac safety guidelines.

## Supplementary Information

Below is the link to the electronic supplementary material.

Supplementary Material 1

## Data Availability

Anonymized data can be available under reasonable request. The corresponding author should be contacted.

## Abbreviations

AF: Atrial fibrillation
AFL: Atrial flutter
APS: Antiphospholipid syndrome
BNP: B‑type natriuretic peptide
CK: Creatine kinase
CKD: Chronic kidney diseases
CK‑MB: Creatine kinase MB isoenzyme
ECG: Electrocardiograph
FPVC: Frequent premature ventricular contraction
FVT: Frequent ventricular tachycardia
HD: Hemodialysis
HIS: Hospital information system
IQR: Interquartile range
LAD: Left atrial diameter
LVEF: Left ventricular ejection fractionPD peritoneal dialysis
PD: Peritoneal dialysis
rhIL‑11: Recombinant human interleukin‑11
rhTPO: Recombinant human thrombopoietin
SLE: Systemic lupus erythematosus
SR: Sinus rhythm
ST: Sinus tachycardia
STROBE: Strengthening the reporting of observational studies in epidemiology
TAFL: Transient atrial flutter
TCM: Traditional chinese medicine
