# Supplementary material for: Cardiac effects of prolonged recombinant human interleukin-11 treatment in severe thrombocytopenia: a retrospective cohort study
Source: BMC Pharmacol Toxicol. 2026 Jul 20;27:115. doi: 10.1186/s40360-026-01188-y (PMC13508329; doi:10.1186/s40360-026-01188-y)
Supplement: Supplementary file 1 [file 40360_2026_1188_MOESM1_ESM.doc]

STROBE Statement—Checklist of items that should be included in reports of ***cohort studies***

|  | Item No | Recommendation | Page/line  No.(Manuscript Chapter Location) | If not reported, give reason |
| --- | --- | --- | --- | --- |
| **Title and abstract** | 1 | (*a*) Indicate the study’s design with a commonly used term in the title or the abstract | Title page (line 1-2) and Abstract (line 35) | Fully reported |
|  |  | (*b*) Provide in the abstract an informative and balanced summary of what was done and what was found | Full Abstract section | Fully reported |
| Introduction | | |  |  |
| Background/rationale | 2 | Explain the scientific background and rationale for the investigation being reported | Introduction full section | Fully reported |
| Objectives | 3 | State specific objectives, including any prespecified hypotheses | Introduction last paragraph(Page4, line 85-90) | Fully reported |
| Methods | | |  |  |
| Study design | 4 | Present key elements of study design early in the paper | Methods, first sentence (page 5, line 94) | Fully reported |
| Setting | 5 | Describe the setting, locations, and relevant dates, including periods of recruitment, exposure, follow-up, and data collection | Methods-Patient Selection(Page5) | Fully reported |
| Participants | 6 | (*a*) Give the eligibility criteria, and the sources and methods of selection of participants. Describe methods of follow-up | Methods-Patient Selection (Page5, line 94-110, screening flowchart Figure1) | Fully reported |
|  |  | (*b*) For matched studies, give matching criteria and number of exposed and unexposed | Not applicable (unmatched cohort) | Not applicable |
| Variables | 7 | Clearly define all outcomes, exposures, predictors, potential confounders, and effect modifiers. Give diagnostic criteria, if applicable | Methods – Diagnosis and evaluations (page 6, lines 111-126: BNP, arrhythmia definition, electrolyte abnormalities, etc.) | Fully reported |
| Data sources/ measurement | 8* | For each variable of interest, give sources of data and details of methods of assessment (measurement). Describe comparability of assessment methods if there is more than one group | Methods – Diagnosis and evaluations (page 6: data from HIS, unified lab/ECG assessments for both groups) | Fully reported |
| Bias | 9 | Describe any efforts to address potential sources of bias | Efforts to address measurement bias and confounding (age, electrolytes, etc.) are described(page 6, lines 112-113); selection bias addressed by consecutive inclusion(page 5, lines 94-96) | Fully reported |
| Study size | 10 | Explain how the study size was arrived at | Methods-Patient Selection (70 initial patients, stepwise exclusion, final n=30; no power calculation performed. Discussion notes limited sample size)(Page 5,line 96-98) | No formal sample size calculation; limited by available data |
| Quantitative variables | 11 | Explain how quantitative variables were handled in the analyses. If applicable, describe which groupings were chosen and why | Methods-Statistical analysis (median/IQR for non-normal, mean±SD for normal; treatment duration stratified by 14 days in Results)(Page 6-7) | Fully reported |
| Statistical methods | 12 | (*a*) Describe all statistical methods, including those used to control for confounding | Methods – Statistical analysis (page 6-7): Mann‑Whitney, Wilcoxon, Kaplan‑Meier, log‑rank, univariate and multivariate Cox regression (adjusted for age, group, etc.) | Fully reported |
|  |  | (*b*) Describe any methods used to examine subgroups and interactions | Methods – Statistical analysis (page 6-8). Subgroup analyses by treatment duration (>14 days) were performed descriptively and presented in the Results section (Table 1). | Formal statistical tests for interaction (e.g., age×group or duration×group) were not performed. This was due to the small sample size (n=30) and the limited number of arrhythmic events (n=8), which precluded reliable fitting of multiplicative interaction terms in the Cox model. The subgroup analyses are considered exploratory. |
|  |  | (*c*) Explain how missing data were addressed | Methods – Statistical analysis (we have added a dedicated sentence: "Missing data were not imputed; all analyses used the available-case approach, with the number of available cases clearly indicated in tables and figure legends."). Also addressed in Discussion – Limitations.(Page 7,line 139-141) | All missing data handling methods are explicitly described. We did not perform multiple imputation or last-observation-carried-forward (LOCF) because the missingness was likely related to clinical routine rather than a random mechanism. Given the small sample size and the exploratory nature of this retrospective study, a complete-case analysis was the most appropriate and transparent approach. |
|  |  | (*d*) If applicable, explain how loss to follow-up was addressed | No loss to follow‑up reported (all included patients had outcome data during hospitalization) | _ |
|  |  | (*e*) Describe any sensitivity analyses | None performed | Not applicable |
| Results | | |  |  |
| Participants | 13* | (a) Report numbers of individuals at each stage of study—eg numbers potentially eligible, examined for eligibility, confirmed eligible, included in the study, completing follow-up, and analysed | Methods-Patient Selection(Page 5) + Figure1 screening flowchart | Fully reported |
|  |  | (b) Give reasons for non-participation at each stage | Figure 1 and text (page 5, lines 96-98): reasons: treatment <5 days or incomplete data | Fully reported |
|  |  | (c) Consider use of a flow diagram | Figure 1 provided | Fully reported |
| Descriptive data | 14* | (a) Give characteristics of study participants (eg demographic, clinical, social) and information on exposures and potential confounders | Table 1 (Results, page 7-8) | Fully reported |
|  |  | (b) Indicate number of participants with missing data for each variable of interest | Table1 indicate BNP available cases; Table2 mark NA for missing BNP records | Fully reported |
|  |  | (c) Summarise follow-up time (eg, average and total amount) | Treatment duration (median, IQR) given in Table 1; follow‑up includes during treatment and one week after discontinuation | Fully reported |
| Outcome data | 15* | Report numbers of outcome events or summary measures over time | Arrhythmia events (8/23) with onset times (Table 2); Figure5 Kaplan-Meier event curves; BNP changes over time (Figures 3‑4) | Fully reported |
| Main results | 16 | (*a*) Give unadjusted estimates and, if applicable, confounder-adjusted estimates and their precision (eg, 95% confidence interval). Make clear which confounders were adjusted for and why they were included | Table 3: univariate and multivariate Cox (adjusted for age, group, etc.). Age was included because p<0.1 in univariate; group included because survival difference significant | Fully reported |
|  |  | (*b*) Report category boundaries when continuous variables were categorized | Continuous variables (age, BNP) were used as continuous in regression(Table 3); treatment duration was categorized only descriptively (>14 days)(Table1) | Not a formal categorical analysis in regression |
|  |  | (*c*) If relevant, consider translating estimates of relative risk into absolute risk for a meaningful time period | Not performed | Not applicable |
| Other analyses | 17 | Report other analyses done—eg analyses of subgroups and interactions, and sensitivity analyses | Subgroup analysis by treatment duration (>14 days, >21 days) described in Results (page 9, lines 192-197) | No formal interaction testing |
| Discussion | | |  |  |
| Key results | 18 | Summarise key results with reference to study objectives | Discussion, first paragraph (page 10) | Fully reported |
| Limitations | 19 | Discuss limitations of the study, taking into account sources of potential bias or imprecision. Discuss both direction and magnitude of any potential bias | Discussion, limitations paragraph (page 15): retrospective design, small sample, single‑center, zero events in rhTPO group affecting Cox model, etc. | Fully reported |
| Interpretation | 20 | Give a cautious overall interpretation of results considering objectives, limitations, multiplicity of analyses, results from similar studies, and other relevant evidence | Discussion, final paragraphs and Conclusions (pages 15-16) | Fully reported |
| Generalisability | 21 | Discuss the generalisability (external validity) of the study results | Discussion, limitations and Conclusions mention limited generalizability due to single‑center and small sample(pages 15) | Fully reported |
| Other information | | |  |  |
| Funding | 22 | Give the source of funding and the role of the funders for the present study and, if applicable, for the original study on which the present article is based | Funding section (page 17-18): grants listed; funders had no role in study design, data collection, analysis, or writing | Fully reported |

*Give information separately for exposed and unexposed groups.

**Note:** An Explanation and Elaboration article discusses each checklist item and gives methodological background and published examples of transparent reporting. The STROBE checklist is best used in conjunction with this article (freely available on the Web sites of PLoS Medicine at http://www.plosmedicine.org/, Annals of Internal Medicine at http://www.annals.org/, and Epidemiology at http://www.epidem.com/). Information on the STROBE Initiative is available at http://www.strobe-statement.org.
